# Causal Effects of Blood Lipid Traits on Inflammatory Bowel Diseases: A Mendelian Randomization Study

**DOI:** 10.3390/metabo13060730

**Published:** 2023-06-07

**Authors:** Ziqin Yao, Feiyu Jiang, Hongbin Luo, Jiahui Zhou, Wanting Shi, Shoufang Xu, Yingying Zhang, Feng Dai, Xinran Li, Zhiwei Liu, Xinhui Wang

**Affiliations:** Department of Blood Transfusion, Sir Run Run Shaw Hospital, School of Public Health, Zhejiang University School of Medicine, Hangzhou 310058, China

**Keywords:** lipid traits, cholesterol, inflammatory bowel diseases, Crohn’s disease, ulcerative colitis, Mendelian randomization

## Abstract

Inflammatory bowel diseases (IBDs), including Crohn’s disease (CD) and ulcerative colitis (UC), have become a global health problem with a rapid growth of incidence in newly industrialized countries. Observational studies have recognized associations between blood lipid traits and IBDs, but the causality still remains unclear. To determine the causal effects of blood lipid traits, including triglycerides (TG), total cholesterol (TC), high-density lipoprotein cholesterol (HDL-C), and low-density lipoprotein cholesterol (LDL-C) on IBDs, two-sample Mendelian randomization (MR) analyses were conducted using the summary-level genome-wide association study (GWAS) statistics of blood lipid traits and IBDs. Our univariable MR using multiplicative random-effect inverse-variance weight (IVW) method identified TC (OR: 0.674; 95% CI: 0.554, 0.820; *p* < 0.00625) and LDL-C (OR: 0.685; 95% CI: 0.546, 0.858; *p* < 0.00625) as protective factors of UC. The result of our multivariable MR analysis further provided suggestive evidence of the protective effect of TC on UC risk (OR: 0.147; 95% CI: 0.025, 0.883; *p* < 0.05). Finally, our MR-BMA analysis prioritized TG (MIP: 0.336; $\hat{\theta }$_MACE_: −0.025; PP: 0.31; $\hat{\theta }$_λ_: −0.072) and HDL-C (MIP: 0.254; $\hat{\theta }$_MACE_: −0.011; PP: 0.232; $\hat{\theta }$_λ_: −0.04) for CD and TC (MIP: 0.721; $\hat{\theta }$_MACE_: −0.257; PP: 0.648; $\hat{\theta }$_λ_: −0.356) and LDL-C (MIP: 0.31; $\hat{\theta }$_MACE_: −0.095; PP: 0.256; $\hat{\theta }$_λ_: −0.344) for UC as the top-ranked protective factors. In conclusion, the causal effect of TC for UC prevention was robust across all of our MR approaches, which provide the first evidence that genetically determined TC is causally associated with reduced risk of UC. The finding of this study provides important insights into the metabolic regulation of IBDs and potential metabolites targeting strategies for IBDs intervention.

## 1. Introduction

Inflammatory bowel diseases (IBDs) mainly consist of two major groups of diseases: Crohn’s disease (CD) and ulcerative colitis (UC). CD can involve any part of the gastrointestinal tract from the oral cavity to the perianal region, while UC is characterized by diffuse persistent colitis extending from the proximal rectum [1]. The pathological and clinical features of the two types of diseases are both distinct and overlapping. IBDs are caused by an overactive immune response to environmental factors in a genetically susceptible host. Whole genome sequencing and other genetic analyses have revealed more than 200 loci associated with IBDs risk [2,3]. In addition, environmental factors, including lifestyle, medication use, and surgery, may play a role in the development of the disease. The distributions of IBDs were initially thought to be limited by race and geography, primarily affecting people of Western European descent [4]. By the late twentieth century, patients suffering from IBDs were identified in all regions of the world [5]. After a rise in the twentieth century, the incidence of IBDs in the western world levels off in this century. However, the incidence of IBDs in the newly industrialized countries of the twenty-first century is increasing [6]. The precise origin of these phenomena remains unknown; however, it can be attributed to the complex interplay between genetics and the environment.

Without prompt and effective treatment, the naturally progressive course of IBDs can advance from presenting mild and infrequent symptoms to causing severe debilitation, requiring surgical intervention and potentially resulting in disability. However, due to technical limitations, current treatment strategies for IBDs focus on symptom relief and aim to prevent all inflammations. The treatment methods currently used, including corticosteroids, immunosuppressants, and biological agents, are ineffective for some patients and may be related to adverse reactions that limit their use [7]. Furthermore, numerous treatment options necessitate long-term parenteral treatment or carry a high risk of severe infections and malignancies [8]. Therefore, prevention of the disease as well as early detection and early elimination of inflammation in the early course of the disease is the best approach to target the disease. Investigating the influence of genetic and environmental factors on the pathogenesis and onset of IBDs may provide insight into risk reduction and symptom alleviation for patients.

Lipids (such as cholesterol and triglycerides) are insoluble in plasma and are transported by binding to circulating lipoproteins [9]. In turn, circulating lipoproteins transport lipids to various tissues for energy utilization, lipid deposition, steroid hormone production, and bile acid formation. Blood contains five major classes of lipoproteins: chylomicrons, very low-density lipoprotein (VLDL), intermediate-density lipoprotein (IDL), low-density lipoprotein (LDL), and high-density lipoprotein (HDL), and each of which carries different amounts of cholesterol and triglycerides [10]. Blood lipid profiles, including total cholesterol (TC), low-density lipoprotein cholesterol (LDL-C), high-density lipoprotein cholesterol (HDL-C), and triglycerides (TG), are widely tested in clinical practice. Lipid profile measurements are currently most commonly used to help determine the risk of cardiovascular disease (CVD) events. Many studies have shown that lipid profiles are closely related not only to metabolic diseases but also to immune and inflammatory disorders [11].

The relationship between blood lipid traits and IBDs has been investigated by prior studies and has yielded varying results. Some case-control studies have observed abnormal blood lipid traits in patients with IBDs [12,13,14,15]. While the findings are conflicting, most studies of individuals with IBDs show a predominant lipid pattern characterized by reduced levels of TC and LDL-C but increased TG and HDL-C levels compared to healthy controls. In addition, the degree of dyslipidemia has been found to correlate with IBDs activity and severity [16]. A study of patients with enterocutaneous fistula showed that IBD was an independent predictor of hypertriglyceridemia [17]. Another observational study found that hypocholesterolemia was more common in patients with IBDs in a hospital population [18]. However, the observational study could not distinguish the causal relationships due to confounding and reverse causality.

Mendelian randomization (MR) can be used to efficiently detect inferences of causal relationships between genetically influenced exposures and diseases. It incorporates genetic instrumental variables (IVs) into traditional causal inference methods and provides a solution to the problem of causality without many of the typical biases that affect the validity of causal inference methods. We conducted MR analysis using large-scale genome-wide association study (GWAS) data on blood lipid traits and IBDs to identify the metabolic causal factor for IBDs prevention and management.

## 2. Method

### 2.1. Mendelian Randomization Analysis Study Design

**Figure 1:**
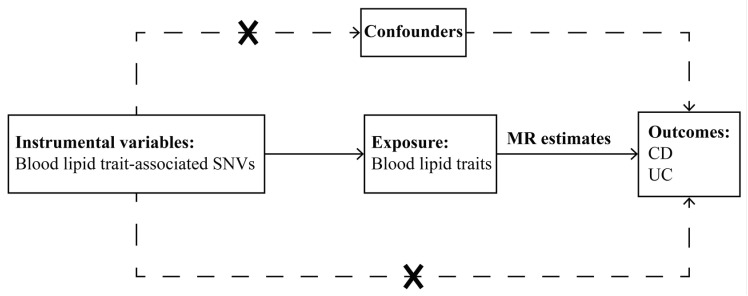
Graphical overview of the two-sample MR study design. Blood lipid trait-associated single nucleotide variations were utilized as instrumental variables to investigate the causal relationship between blood lipid traits and inflammatory bowel diseases. The arrows in the figure denote the assumptions of MR analysis, indicating that the instrumental variable should be associated with the exposure of interest but not related to potential confounders and should influence the outcome only through the exposure. Abbreviations: CD, Crohn’s disease; MR, Mendelian randomization; SNV, single-nucleotide variation; UC, ulcerative colitis.

This study used a two-sample MR design to assess the causal effects of four blood lipid traits and IBDs. The single-nucleotide variations (SNVs) used to demonstrate causal effects in the MR analysis must satisfy three key assumptions: Firstly, the IVs must be closely related to the exposure; Secondly, the IVs are not related to any confounders of the risk factor–outcome association; Thirdly, the IVs do not affect the outcome through any pathway other than the exposure of interest [19]. The fundamental assumptions of MR are depicted in Figure 1.

### 2.2. Data Sources

#### Appropriate IVs for MR Analysis Were Selected from Two Distinct GWASs

Summary-level data of blood lipid traits were obtained from GWASs performed in 45 studies that reported naturally occurring variants in 188,577 individuals of European ancestry and 7898 individuals of non-European ancestry [20]. This GWAS identifies SNVs associated with lipid traits, including TG, TC, LDL-C, and HDL-C. In this study, we used data from a subset that included 37 studies consisting of individuals of European ancestry for subsequent analyses and lessened the chance of population stratification (Supplementary Table S1).

Summary statistics for IBDs were obtained from the International Inflammatory Bowel Disease Genetics Consortium (IIBDGC). The study of IBDs involved 34,652 individuals of European ancestry where the subgroup CD consisted of 5956 patients and 14,927 controls, while the UC subgroup included 6968 patients and 20,464 controls [3]. Diagnoses of CD and UC were based on standard radiological, endoscopic, and histopathological evaluations. Participants were all of European descent. The characteristics of the included cohorts for the GWAS of IBDs are summarized in Supplementary Table S2.

The data sources for this study were all freely available abstract-level information. Ethical approval and written informed consent of the subjects were obtained in all original studies.

### 2.3. Instrument Selection

We carried out a variety of quality control procedures to identify eligible, instrumental SNVs from the GWAS summary data of IBDs, including UC and CD. First, to satisfy the first hypothesis, SNVs associated with corresponding exposures under a genome-wide significance threshold of *p* = 5 × 10^−8^ were selected as initial IVs. For SNVs not available in the IBDs database, proxy SNVs were used based on European population genotype data derived from the 1000 Genomes Project phase 3 (version 5) (r^2^ > 0.8).

To ensure independence, IVs were subjected to a PLINK clustering process. We used a clustering technique with r^2^ < 0.001 and a window size of 10,000 kb to eliminate SNVs associated with significant linkage disequilibrium (LD). Only the SNV with the lower *p* value would be kept among those pairs of SNVs where the r^2^ was higher than the predetermined threshold. Subsequently, the PhenoScanner database (http://www.phenoscanner.medschl.cam.ac.uk/) was used (accessed on 16 February 2023) to find all SNVs associated with potential confounders [21]. All SNVs associated with confounding variables and outcome-related SNVs were eliminated to satisfy the second hypothesis (*p* < 5 × 10^−8^). The threshold of 5 × 10^−8^ is widely recognized as the criterion for declaring genome-wide association significance for common variants with a minor allele frequency of 5% or higher in European ancestry populations [22,23]. This established threshold was based on the International HapMap Consortium’s study in 2005 in which they estimated the number of common independent variants in the European population to be 150 per 500 kilobase pairs (kb) through permutation testing of genotypes in 10 densely genotyped genomic regions and extrapolated to all the genome (~3.3 Gb) and suggested a threshold of 5 × 10^−8^ [24].

In addition, we calculated the *F*-statistic of SNVs for each exposure using the following formula:

$$F=(\frac{R^{2}}{k})/(\frac{\left[1-R^{2}\right]}{\left[n-k-1\right]})$$

where *R*^2^ is the change in exposure explained using the SNV, *n* is the sample size, and *k* is the number of SNVs. The *F*-statistic was used to assess the strength of the IV. *F* < 10 indicates the presence of weak instrumental bias [25].

Finally, the statistical effects of this MR analysis are estimated using a power calculation method based on the Burgess design [26].

### 2.4. MR Estimates

To calculate the causal influence of exposure variables on an outcome, MR analysis employs genetic variations as IVs. In the study, the summary statistics were employed to assess the causative relationships between blood lipid traits (TC, TG, LDL-C, and HDL-C) and IBDs (CD and UC) using various MR approaches. The odds ratios (OR) of CD and UC were calculated per one standard deviation (SD) increment in genetically predicted blood lipid traits.

A variety of univariable MR approaches were employed to assess the causal relationships between blood lipid traits and IBDs, including multiplicative random-effect inverse-variance weighted (IVW), fixed-effect IVW, simple median, weighted median, MR-Egger, and penalized weighted median methods. The fixed-effect IVW method employs a weighted combination of effect estimations obtained from multiple IVs to infer the causal relationship between each blood lipid trait and CD or UC. Meanwhile, the multiplicative random-effect IVW approach accommodates for the presence of heterogeneity across the IVs [27]. Compared to the IVW approaches, the median ratio estimate can tolerate up to 50% of invalid IVs. The weighted median approach utilizes the median effect estimate derived from multiple genetic instruments, whereas the simple median method assumes equal weights for all instruments. On the other hand, the penalized weighted median approach imposes penalty on large deviations from the estimated center of distribution, rendering it useful for data having skewed distribution or outliers that could introduce bias in the estimation of causal effect [28]. The MR-Egger method adopts a regression-based approach to estimate the causal effect of an exposure on an outcome, taking into account the presence of horizontal pleiotropy [29]. The random-effect IVW method was considered as the primary analysis technique, taking into account the existence of several exposures and outcomes. In contrast, the other analytical approaches were used for assessing the robustness of the main findings.

Multivariable MR analysis allows the assessment of the direct effect of exposure factors on outcomes when there is an interaction between exposure factors [30]. Then, conventional linear multivariable MR analysis using IVW methods was performed to assess the direct effects of blood lipid traits on CD and UC.

Finally, a multivariable MR approach based on Bayesian model averaging (MR-BMA) was employed for detecting and prioritizing true risk factors of IBDs from a set of blood lipid traits [31]. In the current study, the method considers all possible combinations of blood lipid traits and generates posterior probabilities (PP) for each model to calculate its marginal inclusion probability (MIP), indicating the probability of it being a causal determinant of disease risk. We also calculated the model-averaged causal estimate ($\hat{\theta }$_MACE_), which represents the average causal effect of the models including lipid indicators. To detect null and influential IVs, Cochran’s *Q* statistic and Cook’s distance (*Cd*) were used to quantify outliers and influential observations, excluding any SNV with a *Q* value greater than 10 or a *Cd* greater than the median of the associated F-distribution [32,33].

### 2.5. Sensitivity Analysis

Heterogeneity of IVs was estimated using Cochran’s *Q* statistic [34]. The intercept obtained from the MR-Egger regression model was employed for assessing the pleiotropy introduced by unknown confounders [35]. To minimize the risk of obtaining false positive results from multiple comparisons, we applied the Bonferroni correction. Following the Bonferroni correction, *p*-values less than 0.00625 (0.05/8, representing 4 exposures and 2 outcomes) were considered statistically significant, while those between 0.00625 and 0.05 were deemed suggestive significant.

Data were analyzed using the TwoSampleMR (version 0.5.6) [36,37] and Mendelian Randomization (0.6.0) [38] packages in the statistical program R (version 4.1.1; the R Foundation for Statistical Computing). All abbreviations used in the manuscript and supplementary tables, their detailed explanations, formula, the R packages employed, and the relevant references for each explanation are shown in Supplementary Note S1.

## 3. Results

### 3.1. Characteristics of SNVs Used as Genetic Instruments

After significance threshold screening, LD clumping, proxy selection, and exclusion of known pleiotropic variants, a total of 109 and 115 independent SNVs were obtained as IVs for CD and UC, respectively (Supplementary Tables S3 and S4). The F-statistics corresponding to individual SNV for CD and UC ranged from 11 to 450, indicating that all SNVs were sufficiently strong (Supplementary Tables S5 and S6). Post hoc power calculations indicated that the sample size included in the current study was large enough (Supplementary Tables S7–S10).

### 3.2. Main Analysis

**Figure 2:**
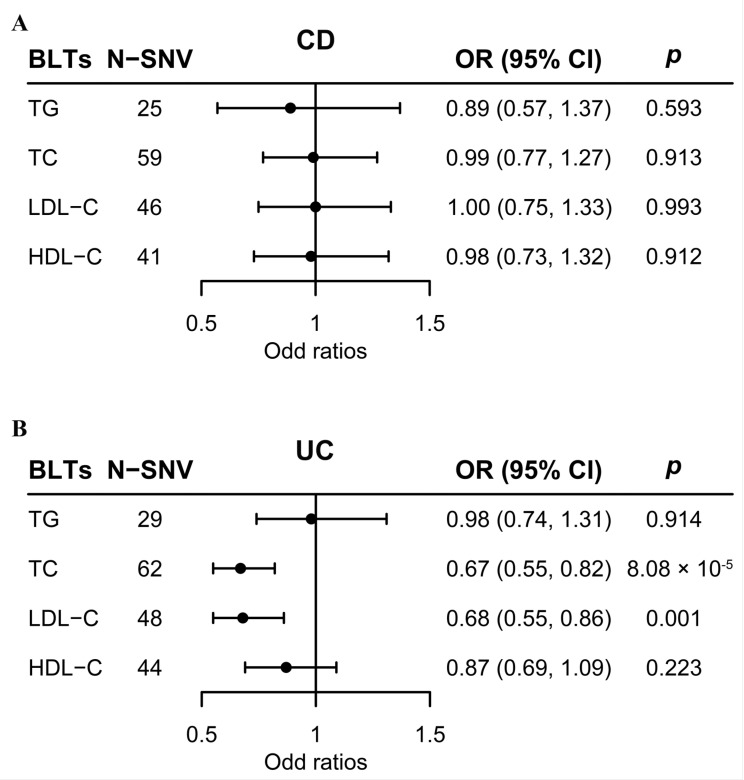
Causal effects of blood lipid traits on CD and UC estimated by univariable MR analysis. (**A**) Association of blood lipid traits and CD in univariable MR analyses. (**B**) Association of blood lipid traits and UC in univariable MR analyses. The estimated ORs reflect the impact of one SD increase in blood lipid traits on CD or UC as determined through multiplicative random-effect inverse-variance weighted analysis. Abbreviations: BLT, blood lipid trait; CD, Crohn’s disease; MR, Mendelian randomization; OR, odds ratio; SD, standard deviation; UC, ulcerative colitis.

Our univariable MR analysis using multiplicative random-effect IVW approach derived distinct results for CD and UC. The results showed no association between blood lipid traits and CD, whereas identified TC (OR: 0.674; 95% CI: 0.554, 0.820; *p* < 0.00625) and LDL-C (OR: 0.685; 95% CI: 0.546, 0.858; *p* < 0.00625) as protective factors for UC (Figure 2).

**Figure 3:**
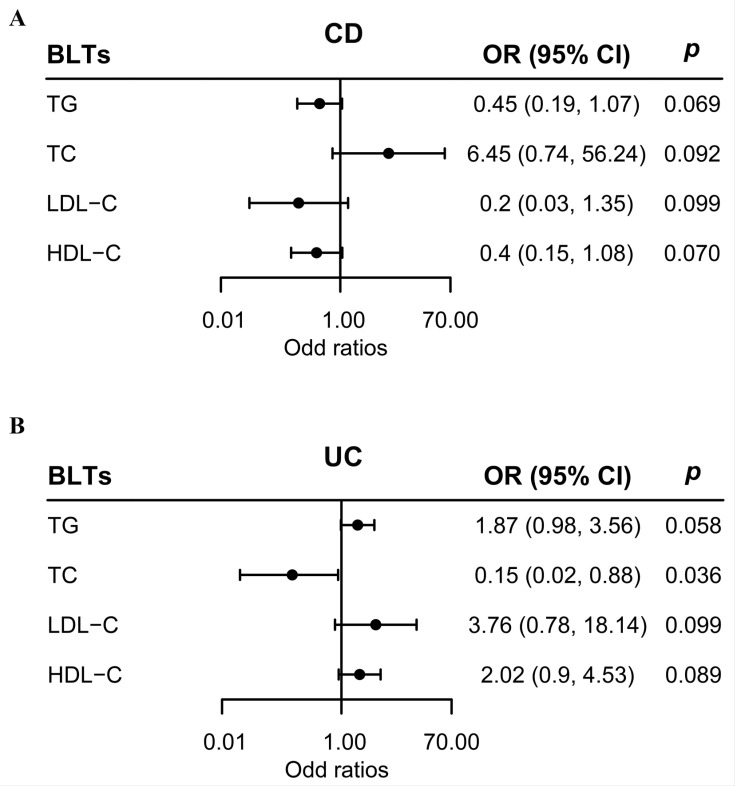
Causal effects of blood lipid traits on CD and UC estimated by multivariable MR analysis. (**A**) Association of blood lipid traits and CD in multivariable MR analyses. (**B**) Association of blood lipid traits and UC in multivariable MR analyses. The estimated ORs reflect the impact of one SD increase in blood lipid traits on CD or UC, as determined through multiplicative random-effect inverse-variance weighted analysis. Abbreviations: BLT, blood lipid trait; CD, Crohn’s disease; MR, Mendelian randomization; OR, odds ratio; SD, standard deviation; UC, ulcerative colitis.

The subsequent multivariable MR analysis using multiplicative random-effect IVW approach derived consistent results. No causal factor was detected for CD in multivariable MR analysis (Figure 3A). The result of multivariable MR for UC provided suggestive evidence for the protective effect of TC (OR: 0.147; 95% CI: 0.025, 0.883; *p* < 0.05) on UC (Figure 3B), which was directionally consistent with our univariable MR result.

Furthermore, the non-linear MR-BMA approach was employed for detecting the best models of CD and UC (Table 1 and Table 2). TG (MIP: 0.336; $\hat{\theta }$_MACE_: −0.025; PP: 0.31; $\hat{\theta }$_λ_: −0.072) and HDL-C (MIP: 0.254; $\hat{\theta }$_MACE_: −0.011; PP: 0.232; $\hat{\theta }$_λ_: −0.04) were prioritized as best models for CD, whereas TC (MIP: 0.721; $\hat{\theta }$_MACE_: −0.257; PP: 0.648; $\hat{\theta }$_λ_: −0.356) and LDL-C (MIP: 0.31; $\hat{\theta }$_MACE_: −0.095; PP: 0.256; $\hat{\theta }$_λ_: −0.344) were prioritized for UC with PP larger than 0.02 and MIP larger than 0.25 (Table 2). The *Q* statistic and *Cd* for each IV are shown in Supplementary Tables S11–S14.

### 3.3. Sensitivity Analysis

The PhenoScanner database was employed for known pleiotropic variants exclusion. The phenotypes associated with the selected genetic IVs for CD and UC are shown in Supplementary Table S15.

The fixed-effect IVW, simple median, weighted median, MR-Egger, and penalized weighted median methods in the univariable MR analysis derived directionally consistent results as the multiplicative random-effect IVW method (Supplementary Tables S16 and S17).

Finally, no evidence of horizontal pleiotropy was found according to the MR-Egger regression intercept (*p* > 0.05) (Supplementary Tables S18 and S19). Furthermore, the Cochran’s *Q* statistic indicated no heterogeneity across the IV estimates determined with the MR-Egger and IVW methods for UC but indicated relatively high heterogeneity for CD (Supplementary Tables S20 and S21).

## 4. Discussion

In this study, we performed a comprehensive MR analysis using data retrieved from two large-scale GWAS to investigate the causal relationship between blood lipid traits and IBDs, which fills a gap in the field. No evidence of a causal relationship between blood lipid traits and CD was found in our MR analyses. In contrast, the causal effect of TC on UC was identified in our univariable MR analysis and showed directional consistency across all analytical MR approaches used in this study.

Previous studies have recognized high lipid levels and obesity were associated with many diseases, including inflammatory bowel disease (IBDs). A meta-analysis of 255 studies demonstrated an association between dietary fat, cholesterol, fatty acids, and the risk of IBDs [39]. An observational study involving 1598 children with IBDs indicated that around 20% of those with CD and 33% of those with UC were overweight or obese. Obese children with IBDs may experience a more severe disease progression [40]. These results suggest that improper dietary lipid composition or abnormal anthropometric variables may exacerbate IBDs.

Interestingly, patients with active chronic inflammatory diseases have lower levels of TC, HDL-C, and LDL-C compared to the general population or patients with disease in remission [41]. In systemic lupus erythematosus, rheumatoid arthritis, and sepsis, this phenomenon, known as dyslipidemia, is prevalent [42,43,44,45]. Multiple studies have proposed that patients with CD and UC have lower TC and LDL-C levels than controls [46,47,48]. A case-control study found a significant association between low TC and LDL-C levels and active UC and CD, indicating a systemic inflammatory state [46]. A retrospective study involving 701 patients with IBDs compared to the general population found that patients experience a decreased occurrence of TC and LDL-C and an increased incidence of HDL-C and elevated TG. In addition, a population-based observational study examined the plasma lipids in IBDs and found that plasma TC and LDL-C were slightly lower in IBDs patients compared to individuals from the general population [48]. Thus, these association studies have linked blood lipid profiles, especially TC and LDL-C to IBDs status.

Recently, in a prospective study conducted in IBDs patients using prednisone and tofacitinib induction therapy, it was found that remission in clinical scores including the Harvey Bradshaw Index for CD and Simple Clinical Colitis Activity Index for UC were significantly correlated with an increase in serum TC level [49]. Other clinical investigations of the efficacy of IBDs medications have also revealed that the blood cholesterol profiles, especially TC and LDL-C, were significantly increased in IBDs patients during and after treatment compared to before treatment, irrespective of the types of medications used [50,51,52,53]. In a systematic review and meta-analysis involving 1663 patients across 11 studies, corticosteroid and tofacitinib induction therapy resulted in a significant increase in TC [50]. A clinical trial showed that upadacitinib, a selective Janus kinase 1 (JAK1) inhibitor, significantly increased TC, HDL-C, and LDL-C levels in IBDs patients who took the medication and experienced clinical remission [51]. To sum up, the elevation of blood lipids, particularly TC and LDL-C, has been found to have a positive correlation with improved clinical amelioration and better prognosis for IBDs patients.

Many observational studies on UC provide evidences of correlations between disease severity and blood lipid profile. In a prospective study, TC was found to be significantly elevated in UC patients with mucosal healing compared to those without mucosal healing [16]. Another meta-analysis of 3 cohorts involving 1157 UC patients showed that the tofacitinib group had higher levels of TC, HDL-C, and LDL-C accompanied by decreased high-sensitivity C-reactive protein (hs-CRP) level compared to the placebo group. Notably, this reverse correlation between the cholesterol profiles and hs-CRP was observed not only in the tofacitinib group but also in the placebo group, which may implicate a more general pathophysiological effect [52]. The finding of these studies implicated that relatively high levels of cholesterol profiles, especially TC and LDL-C, are related to a good prognosis. Consistent with previous studies, our MR analysis further determined the causality between blood cholesterol and UC risk.

CD studies exhibit a similar pattern of association where CD patients in remission post-bowel resection have higher levels of TC, HDL-C, and LDL-C compared to those with recurrent CD [54]. Additionally, the results of a study comprising of 868 CD patients indicate high levels of TG, low levels of HDL-C, and diabetes as risk factors for CD-related hospitalizations [55]. Although there are evidences of an association between blood lipid profile and CD in some clinical studies, the results of this MR study did not find a significant causal effect of blood lipid profile on CD.

Although previous observational studies have implicated the reverse correlation between systemic cholesterol and intestinal inflammation, the clues for understanding its potential mechanism are still limited. We speculate that multiple pathways may be involved in this process. Firstly, the intestinal enterocyte is not only the major cell for dietary cholesterol absorption but also the secondary large source of de novo synthesized cholesterol besides the hepatocyte. The decreased blood cholesterol level may prompt enterocyte to accelerate the synthesis of cholesterol, leading to excessive consumption of ATP. Indeed, the levels of cholesterol, cholesterol sulfate, and genes involved in cholesterol biosynthesis are significantly elevated in inflamed tissues from UC patients compared to healthy controls. Furthermore, supplementation of cholesterol sulfate alleviated dextran sulfate sodium (DSS)-induced UC in mice [56]. Secondly, hypocholesterolemia may interfere with the synthesis of steroid hormones and, hence, cause immune imbalance and intestinal dysfunction. Cholesterol is an important lipid that takes part in cell membrane structure formation and is also a critical component for synthesizing numerous hormones and biomolecules in the human body [57,58]. For example, glucocorticoids, such as cortisol, are important steroid hormones synthesized from cholesterol and possess anti-inflammatory properties. IBDs are common gastrointestinal disorders, and glucocorticoids are commonly used for moderate-to-severe IBDs intervention [59]. Therefore, high blood cholesterol levels may facilitate the physiological synthesis of glucocorticoids, which could help suppress the occurrence and progression of IBDs. Thirdly, cholesterol can affect the bile acid metabolism, which could further regulate gut microbiota, thereby affecting the health status of intestine. Bile acids have become a key class of microbiota-related metabolites that are disrupted in patients with IBDs. In recent years, metabolomics studies have shown that there is a sustained defect in bile acid metabolism in patients with IBDs, characterized by increased primary bile acids and decreased secondary bile acids, which suggests impaired bacterial conversion of primary to secondary bile acids [60,61,62]. This pattern can be due to a disruption in the gut microbiota, such as decreased levels of bacteria responsible for the conversion of primary to secondary bile acids or an increase in bacteria that deconjugate and dehydroxylate secondary bile acids. This may have implications for the pathogenesis and management of IBDs. The liver requires cholesterol from the bloodstream to synthesize bile acids, and thus, hypercholesterolemia provides sufficient substrate which plays a dual role in promoting the absorption of fat-soluble substances in the intestine as well as influencing the structure and function of intestinal flora either directly or indirectly [61,63]. Although the etiology of IBDs is unknown, current research has established that the gut microbiota also plays a crucial role in the development and progression of ulcerative colitis [64,65,66]. Thus, blood cholesterol may regulate the composition of bile acids and subsequently impact the gut microbiota, which can in turn affect the pathogenesis of IBDs. In summary, the excessive consumption of ATP for accelerated cholesterol synthesis, the disturbance of immune response due to the altered steroid hormones metabolism, and the disruption of gut microbiota caused by altered composition of bile acids may collectively contribute to the pathological development of UC. Although these hypotheses require further investigation, it highlights the multiple important functions of cholesterol in the human body and underscores the necessity of controlling cholesterol levels in a reasonable manner.

In clinical practice, blood lipid traits serve as indicators of health status and lipid-related diseases. Our MR analyses use both standard linear regression and non-linear Bayesian model averaging approaches, providing the most up-to-date and thorough evidence on the causal links between blood lipid traits and IBDs. The findings of this study provide new insights into the potential use of blood lipid traits in the prevention and management of IBDs. Blood lipid traits could be used as targets for intervention in managing IBDs as they offer a minimally invasive, cost-effective, and readily available method of treatment. For prevention and triage, clinicians should take the serum lipid status, especially the TC or LDL-C levels, into account for UC. Gastrointestinal symptoms that occur in patients with low levels of TC or LDL-C should be identified as they are often considered as complaints of hepatobiliary disorders but not as an early clinical manifestation of UC. Failure to distinguish between these conditions may lead to delay in diagnosis. For disease management, incorporating the lipid profile test and involved appropriate intervention for low TC and LDL-C levels as part of routine practice may help to optimize the clinical strategy for UC and improve prognosis.

Despite the significant findings, this study has some limitations. One limitation is that the study only included exposures related to a limited number of lipid indicator categories. A comprehensive analysis of various exposures may lead to more interesting findings. However, the routine blood lipid profile detected in clinical practice was included in this study, making it more convenient for clinical application. The study was limited to populations of European ancestry due to the availability of genetic data, which may limit the generalizability of the results. Heterogeneity was found in the univariable MR analysis for the causality between blood lipid traits and CD, which could also produce false negative results. Furthermore, the findings of this study are based on the analyses of statistics from GWAS database and have yet to be validated in population. To obtain primary evidence, it is essential to conduct subsequent investigations, such as perspective cohort studies or randomized controlled trials. Through these additional studies, it will be possible to confirm the validity and generalizability of the conclusions drawn from this study and to strengthen the overall scientific rigor of the findings.

## 5. Conclusions

Using MR approaches, we examined the causality between blood lipid traits and IBDs. Our univariable MR analysis identified TC and LDL-C as protective factors of UC (*p* < 0.00625). The following multivariable MR analysis produced suggestive evidence for the protective effect of TC on UC (*p* < 0.05). Finally, the MR-BMA analysis further prioritized TC and LDL-C as the top two ranked causal factors of UC and prioritized TG and HDL-C as the top two ranked causal factors of CD. Notably, TC was the only protective factor consistently associated with UC across all univariable MR, multivariable MR, and MR-BMA approaches. In summary, our result provided the first evidence that genetically determined TC is causally associated with reduced risk of UC. As serum TC level is also associated with the risk of cardiovascular diseases, further studies are needed to clarify the optimal range of serum TC as well as the mechanism underlying the protective effect of TC on UC, thereby providing new insights regarding clinical strategies for IBDs prevention and management.

## Figures and Tables

**Table 1:** Ranking of risk factors and models (sets of risk factors) for CD ^a^.

(A) Model Averaging for Risk Factors			
Ranking by MIP	Risk Factor	MIP	$\hat{\theta }$_MACE_
1	TG	0.336	−0.025
2	HDL-C	0.254	−0.011
3	LDL-C	0.233	−0.006
4	TC	0.226	−0.006
(B) The 10 Best Individual Models			
Ranking by PP	Model	PP	$\hat{\theta }$ _λ_
1	TG	0.31	−0.072
4	HDL-C	0.232	−0.04
3	LDL-C	0.209	−0.027
2	TC	0.202	−0.027
2,3	TG, LDL-C	0.011	−0.02, −0.009
1,4	TC, HDL-C	0.01	−0.129, −0.09
1,3	TG, LDL-C	0.007	−0.066, −0.014
1,2	TG, TC	0.007	−0.066, −0.015
2,4	TC, HDL-C	0.005	−0.017, −0.034
3,4	LDL-C, HDL-C	0.005	−0.023, −0.037

^a^ Results were generated using the MR-BMA approach. A total of 4 measurable circulating lipid traits genetically instrumented by 109 SNVs were assessed as risk factors. All risk factors and the 10 best individual models were presented. A negative causal estimate ($\hat{\theta }$_MACE_ or $\hat{\theta }$_λ_) indicates a protective effect as suggested by the model, whereas a positive value indicates a risk factor. $\hat{\theta }$_λ_ is the causal effect estimate for a specific model, and $\hat{\theta }$_MACE_ is the model averaged causal effect of a risk factor. CD, Crohn’s disease; HDL-C, high-density lipoprotein cholesterol; LDL-C, low-density lipoprotein cholesterol; MIP, marginal inclusion probability; MR, Mendelian randomization; MR-BMA, MR based on Bayesian model averaging; PP, posterior probability; SNV, single-nucleotide variation; TC, total cholesterol; TG, triglycerides.

**Table 2:** Ranking of risk factors and models (sets of risk factors) for UC ^a^.

(A) Model Averaging for Risk Factors			
Ranking by MIP	Risk Factor	MIP	$\hat{\theta }$_MACE_
1	TC	0.721	−0.257
2	LDL-C	0.31	−0.095
3	TG	0.033	0.003
4	HDL-C	0.031	−0.002
(B) The 10 Best Individual Models			
Ranking by PP	Model	PP	$\hat{\theta }$_λ_
2	TC	0.648	−0.356
3	LDL-C	0.256	−0.344
2,3	TC, LDL-C	0.033	−0.334, −0.025
1,2	TG, TC	0.022	0.096, −0.374
2,4	TC, HDL-C	0.016	−0.347, −0.032
3,4	LDL-C, HDL-C	0.01	−0.329, −0.118
1,3	TG, LDL-C	0.008	0.083, −0.359
4	HDL-C	0.003	−0.167
1,2,3	TG, TC, LDL-C	0.001	0.097, −0.349, −0.028
1	TG	0.001	−0.012

^a^ a Results were generated using the MR-BMA approach. A total of 4 measurable circulating lipid traits genetically instrumented by 115 SNVs were assessed as risk factors. All risk factors and the 10 best individual models were presented. A negative causal estimate ($\hat{\theta }$_MACE_ or $\hat{\theta }$_λ_) indicates a protective effect as suggested by the model, whereas a positive value indicates a risk factor. $\hat{\theta }$_λ_ is the causal effect estimate for a specific model, and $\hat{\theta }$_MACE_ is the model averaged causal effect of a risk factor. HDL-C, high-density lipoprotein cholesterol; LDL-C, low-density lipoprotein cholesterol; MIP, marginal inclusion probability; MR, Mendelian randomization; MR-BMA, MR based on Bayesian model averaging; PP, posterior probability; SNV, single-nucleotide variation; TC, total cholesterol; TG, triglycerides; UC, ulcerative colitis.

## Data Availability

The data presented in this study are available in article and supplementary material. The publicly available summary data on inflammatory bowel diseases and blood lipid traits were provided by the International Inflammatory Bowel Disease Genetics Consortium (IIBDGC) and the Global Lipids Genetics Consortium (GLGC), respectively.

## Supplementary Materials

The following supporting information can be downloaded at: https://www.mdpi.com/article/10.3390/metabo13060730/s1, Table S1: Characteristics and statistics of studies on blood lipid traits; Table S2: Characteristics and statistics of studies included based on the IIBDGC; Table S3: Characteristics of genetic instruments for circulating lipid traits in CD with 109 SNVs; Table S4: Characteristics of genetic instruments for circulating lipid traits in UC with 115 SNVs; Table S5: The *R^2^* & *F* of SNVs in CD in univariable Mendelian randomization analyses; Table S6: The *R^2^* & *F* of SNVs in UC in univariable Mendelian randomization analyses; Table S7: The power of blood lipid traits in CD in univariable Mendelian randomization analyses; Table S8: The power of blood lipid traits in UC in univariable Mendelian randomization analyses; Table S9: The power of blood lipid traits in CD in multivariable Mendelian randomization analyses; Table S10: The power of blood lipid traits in UC in multivariable Mendelian randomization analyses; Table S11: Model diagnostics for MR-BMA analysis using q statistic for the instruments based on the best individual models with PP > 0.02 in CD; Table S12: Model diagnostics for MR-BMA analysis using q statistic for the instruments based on the best individual models with PP > 0.02 in UC; Table S13: Model diagnostics for MR-BMA analysis using *Cd* for the instruments based on the best individual models with PP > 0.02 in CD; Table S14: Model diagnostics for MR-BMA analysis using *Cd* for the instruments based on the best individual models with PP > 0.02 in UC; Table S15: Association phenotypes of instrumental variables for CD and UC; Table S16: Association between genetically instrumented blood lipid traits and CD using the 109 selected SNVs; Table S17: Association between genetically instrumented blood lipid traits and UC using the 115 selected SNVs; Table S18: Pleiotropy in CD; Table S19: Pleiotropy in UC; Table S20: Heterogeneity in CD; Table S21: Heterogeneity in UC; Note S1: Explanations of abbreviations used.

## Abbreviations

95% CI: 95% confidence interval; *Cd*, Cook’s distance; CD, Crohn’s disease; CVD, cardiovascular disease; DSS, dextran sulfate sodium; GLGC, the Global Lipids Genetics Consortium; GWAS, genome-wide association study; HDL, high-density lipoprotein; HDL-C, high-density lipoprotein cholesterol; hs-CRP, high-sensitivity C-reactive protein; IBDs, inflammatory bowel diseases; IDL, intermediate-density lipoprotein; IIBDGC, the International Inflammatory Bowel Disease Genetics Consortium; IV, instrumental variable; IVW, inverse variance weighted; LD, linkage disequilibrium; LDL, low-density lipoprotein; LDL-C, low-density lipoprotein cholesterol; MACE, the model-averaged causal estimate; MIP, marginal inclusion probability; MR, Mendelian randomization; MR-BMA, MR based on Bayesian model averaging; OR, odds ratio; PP, posterior probability; SD, standard deviation; TC, total cholesterol; TG, triglycerides; SNV, single-nucleotide variation; UC, ulcerative colitis; VLDL, very low-density lipoprotein.
